# Impact of self-determination theory in a physiotherapeutic training: A pilot-study on motivation for movement of obese adolescents

**DOI:** 10.1007/s00508-021-01849-4

**Published:** 2021-04-09

**Authors:** Johanna Strempfl, Teresa Wutzl, Didem Ün, Susanne Greber-Platzer, Mohammad Keilani, Richard Crevenna, Alexandra Thajer

**Affiliations:** 1 Department of Health, St. Pölten University of Applied Sciences, St. Pölten, Austria; 2 Department of Pediatrics and Adolescent Medicine, Medical University of Vienna, Währinger Gürtel 18–20, 1090 Vienna, Austria; 3 Department of Physical Medicine, Rehabilitation and Occupational Medicine, Medical University of Vienna, Vienna, Austria

**Keywords:** Intervention, Obesity, Physical exercise, Prevention, Therapeutic group training

## Abstract

**Background:**

This study determined to what extent the underpinning of physiotherapeutic interventions with the evidence-based motivational psychological concept of the self-determination theory (SDT) by Ryan and Deci can increase motivation and enjoyment of movement in obese adolescents.

**Methods:**

In this study 12 obese adolescents aged 14–18 years were offered a targeted group-specific sports program including a home exercise program of 8 weeks. The group leaders were trained in the SDT and supported to integrate motivational aspects. A SDT-based questionnaire by Kohake and Lehnert was used to evaluate motivational interventions.

**Results:**

In total, seven (58%) patients finished the study. In the before-after comparison there were little changes in motivation. Results showed that contrary to expectations the motivation of the obese adolescents to move and to participate in the study was generally high. In the study, more internalized forms of motivation dominated, the highest quality form of motivation.

**Conclusion:**

Digital technologies could be a successful way to further increase motivation and compliance of our target group. This MotiMove study is a basis for future research programs and empower physiotherapists and movement experts to develop and implement training programs for obese adolescents and children.

**Supplementary Information:**

The online version of this article (10.1007/s00508-021-01849-4) contains supplementary material, which is available to authorized users.

## Introduction

The global prevalence of overweight and obesity has doubled over the past 40 years and is increasing worldwide among children and adolescents [1, 2]. Obesity leads to an increased risk of noncommunicable diseases, such as cardiovascular diseases, diabetes and cancer, and is associated with degenerative muscle and joint diseases as well as psychosocial, psychological and psychiatric consequences [2–5].

In addition to the genetic predisposition, environmental factors, such as eating habits, physical activity, socioeconomic factors, environmental and cultural aspects play a central role in the multifactorial genesis [6, 7]. This results in the relevance of multimodal therapy with the three basic components of nutrition, exercise and behavioral therapy [6]. Social physical fear, body shame, stigmatization and harassment of adolescents with an increased body mass index (BMI) have a negative impact on sport commitment [8–10]. The poor athletic performance leads to frustration, demotivation and further withdrawal from the athletic activity, and the joy of movement is lost [11].

The positive effects of exercise programs for obese adolescents on a physical and psychological level have been confirmed by numerous studies [12–14]. Therefore, under the guidance of physiotherapists they represent a suitable therapeutic measure for this target group. In addition to specialist knowledge of the musculoskeletal system, psychosocial strategies for behavior change must be considered as clinical competencies in physiotherapy. For this purpose, Deci and Ryan’s psychological concept of the self-determination theory (SDT) was scientifically examined [15]. According to the SDT, experiencing autonomy, competence and social integration is an essential prerequisite for the development of intrinsic motivation. Intrinsic motivation is the most valuable form of motivation, which is expressed in completely internalized and self-determined actions. In contrast, the extrinsic motivation is an externally determined motivation. In between are further gradations on a continuum, such as the introjected motivation, which is characterized by self-control and the identified motivation, in which the importance of behavior for oneself is already conscious [16]. Autonomy means the possibility to self-regulate one’s own actions, competence describes the ability to manage challenges, and social integration means having a close connection to other individuals and being a recognized member of a social group [16, 17]. Promotion of motivation represents the advancement of these three basic psychological needs. A motivational process to increase physical activity with the support of coaches is associated with the prevention of obesity in adolescents [18]. Positive and clear communication with adolescents and the well-being in a group make a decisive difference with respect to the development of intrinsic motivation for movement [19]. Constructive feedback, active listening, humor and clarifying the relevance of the learning content play an essential role [8]. The evidence of the practical implementation of the SDT was examined in different fields [16, 20, 21]. So far, only a few study results have been available for physiotherapy regarding the practical implementation of the SDT and data for adolescents with obesity are missing [15, 22].

The aim of this pilot study was to examine the effect of the implementation of motivational aspects based on the SDT in an 8‑week physiotherapeutic group training combined with a home exercise program that aimed to motivate obese adolescents to engage in physical activity. The hypothesis was that the motivation of the obese adolescents to move is generally low and that motivation to participate in exercise programs can be improved by the implementation of the SDT in physiotherapeutic group training.

## Material and methods

### Study design and ethics

The pilot study was an intervention in a pre/post design to determine the motivation for movement (MotiMove study) of obese adolescents. The ethics committee of the Medical University of Vienna approved the study (EK Nr: 1572/2019). Parents as legal representatives and their children gave written informed consent before they participated in the study.

### Patients

A total of 12 patients, aged 14–18 years, with a BMI defined as > 90th age-based percentile [23] from the outpatient clinic of obesity, lipometabolic disorder and nutritional medicine, Department of Pediatrics and Adolescent Medicine, Medical University of Vienna were included in this study from October 2019 to December 2019. Patients with pregnancy, syndromes (e.g. Prader-Willi syndrome), chronic joint diseases, neuromotor and neurological diseases, and operations on the lower extremity in the last 6 months were excluded.

### Measurements

A three-part questionnaire to evaluate the integration of motivational aspects and their effects was created. The first part of the questionnaire concentrated on basic motivation to participate in the study, frequency of sporting activity in addition to the study and sporting interests and was completed after the third unit of eight training sessions. The third training session was selected for the first measurement date; to ensure that the study population had gained experience with the group training.

The second part of the questionnaire measures the primary parameter of the change in motivation to move in the before-after comparison and is based on the valid questionnaire for children in sports to measure the self-concordance of sport-related and exercise-related goals by Kohake and Lehnert [24]. This SDT-based tool divides motivation into four subcategories: the extrinsic, introjected, identified and intrinsic motivation. The second part of the questionnaire was completed after the third and seventh training session to compare the quality of motivation to move.

After the seventh training session, the participants had the opportunity to give feedback. This third part of the questionnaire was designed by the authors and contains questions about the home exercise program. An external coach from a local Viennese sports association “Sportunion Wien” designed the eighth training session. Therefore, this last session did not fall into the period under investigation.

### Intervention

There were five group leaders who were trained on the theoretical background of the SDT and the implementation of the motivational aspects in physiotherapeutic group training. The three female and two male group leaders were students at the Physiotherapy Degree Program of St. Pölten University of Applied Sciences. The obese adolescents were educated on the inclusion of motivation encouragement from an external to internal motivation. The training was adapted to the needs of the patients and different training materials, degrees of severity and intensity of the training were carried out in order to create a varied and customized training for the obese adolescents. A structured training session, building awareness on purpose of physical exercise and a positive feedback should improve self-confidence and competence. Social integration was promoted by a respectful interaction of group leaders with the obese adolescents, addressing participants by their first names and partner exercises (e.g. ball games, giving “high five” when passing by, playing tag, coordination exercises etc.). The group leaders developed a movement program tailored to the target group. Adolescents with obesity suffer from premature knee and hip joint symptoms [3]. The aim of the training program was to minimize the loads on these structures through leg axis training, to strengthen the muscles surrounding the joints, and to activate the entire body. Strength endurance training with stabilization exercises to relieve the lower extremities has been integrated. In the exercise program, individual emphasis was placed on training intensity in the low-intensity range.

Two group leaders performed the exercise program once a week for 60 min per session for a study period of 8 weeks at the Department of Physical Medicine, Rehabilitation and Occupational Medicine at the Medical University of Vienna. In addition, the participants had the opportunity to carry out the approximately 15–20min home exercise program at least once a week at home (supplementary material). With the support of coordinated training documents, the patients repeated some of the exercises from the group lessons. The local Viennese sports association “Sportunion Wien” was invited to the last exercise session, which offered a selected exercise program with a focus on coordinative ball sports training based on the individual sports preferences of our study group. All programs offered were free of charge. This should minimize the hurdle to continue exercising autonomously and provide adolescents with obesity programs a low-threshold opportunity to register independently for a further exercise program.

### Statistical analysis

Data analysis was carried out using the software SPSS (version 24.0, SPSS Corp., Chicago, IL, USA). It was examined whether and to what extent the type of encouragement changes on a continuum from controlled and extrinsic to an autonomous and intrinsic form. Baseline data were presented in mean and standard deviation or median and range. For the primary hypothesis (ordinally scaled, 4‑point Likert scale), the results were described as median and interquartile range (IQR) and graphically represented with bar charts. By evaluating the frequencies, the data were checked for changes in the before-after comparison. The 30 items of the MotiMove questionnaire part 2 [24] can be assigned to the different categories. A per-protocol (PP) analysis, defined as participation in a minimum of 60% (5 out of 8) of the training sessions, was carried out. Due to the small sample size, a normal distribution could not be demonstrated.

## Results

### Study population

In total 12 obese adolescents were recruited for the MotiMove study, 7 (58%) participants finished the study and participated in at least 60% of the training, and 5 participants did not reach the minimum attendance (caused by school stress, poor time management, and other obligations). The 7 study participants (57% male) had a mean age of 16.1 ± 1.5 years, body height of 166.8 ± 10.4 cm, body weight of 104.8 ± 16.2 kg, BMI of 37.6 ± 4.3 kg/m^2^, and a mean body fat mass of 53.2 ± 16.3 kg (Table 1). All participants had a migrant background.

**Table 1:** Baseline characteristics of the study population

Characteristics	Patients (*n* = 7)
Male (%)	4 (57%)
Age (years)	17 (14–18)
SBP (mm Hg)	132 (111–148)
DBP (mm Hg)	74 (65–104)
Pulse rate (beats/min)	88 (55–97)
Height (cm)	163 (157–183)
Weight (kg)	111 (78–123)
BMI (kg/m^2^)	37 (31–45)
Abdominal circumference (cm)	114 (101–128)
Hip circumference (cm)	123 (106–131)
Mid upper-arm circumference (cm)	39 (34–40)
BFP (%)	47 (36–58)
FM (kg)	51 (28–79)
FFM (kg)	50 (47–79)
TBW (kg)	37 (34–58)

Results are given as median and range or as number of subjects (%)

*SBP* systolic blood pressure, *DBP* diastolic blood pressure, *BMI* body mass index, which was calculated as body weight divided by body height squared using the formula (weight in kg) /(height in m)^2^. Body composition was measured with bioelectrical impedance analysis (TANITA, Type BC-418MA, Tokyo, Japan). *BFP* body fat percentage, *FM* fat mass, *FFM* fat free mass, *TBW* total body water

### Basic motivation to move

Multiple answers were possible about the basic motivation to participate in the study and to perform physical activity. Therefore, the statistical evaluation was carried out in form of defined multiple answer sets as dichotomies. The highest response frequency related to the intrinsic and the identifying forms of motivation. The question “I decided to take part in the study and to be physically active …” was answered six times with “because it’s good for me” (identified motivation), followed by five times with “because it’s just fun” (intrinsic motivation). “Because others say I should be active in sports” (extrinsic motivation) was ticked once as well as “because I think that sometimes you have to force yourself to do something” (introjected motivation) (Table 2).

**Table 2:** Response frequencies of the basic motivation

Basic motivation	Response frequencies (*N*)	%
Extrinsic motivation	1	7.7
Introjected motivation	1	7.7
Identified motivation	6	46.1
Intrinsic motivation	5	38.5
*Total*	*13*	*100*

The sport activity outside the study revealed that additional physical activity was performed in 71% of the participants and 29% practiced sport more than twice a week with a high preference for ball sports. Back pain and the lack of success were the reasons that 29% did not perform any exercise.

### Results of the MotiMove questionnaire part 2

**Fig. 1:**
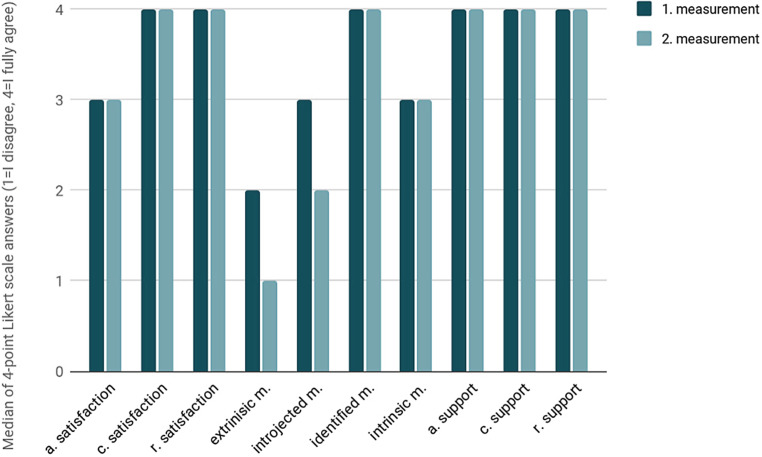
Overall results of the MotiMove-questionnaire part 2 *a* autonomy, *c* competence, *r* relatedness, *m* motivation

On a 4-point Likert scale, the participants selected the answers to the statement from 1 = “I disagree” to 4 = “I fully agree”. The median was calculated from the 4‑point Likert scale answers. The satisfaction of basic needs for autonomy, competence and social integration did not change in the before-after comparison. Satisfaction of autonomy with a median of 3 showed that this factor was not completely exhausted. The satisfaction of competence and social integration with a median of 4 each was very high. The results of the data evaluation indicated a low extrinsic motivation with a median of 2 for the first measurement, which decreased to 1 for the second measurement. The introjected motivation also improved from a median of 3 to 2. The highest median of 4 was found in the identified motivation, which did not change between the first and the second measurement. With a median of 3, the intrinsic motivation (highest quality form of motivation), is the aim of every motivation promotion [16] in both measurements. When evaluating the promotion of basic needs, there were no changes between the two measurements in all three categories. With a continuous median of 4, there was a high level of teaching quality in terms of motivational support. The overall results of MotiMove questionnaire part 2 are shown in the diagram (Fig. 1). The feedback on the MotiMove exercise units was consistently positive. The participants said they had fun in the training and found the lessons professional.

### Results of the MotiMove questionnaire part 3

**Fig. 2:**
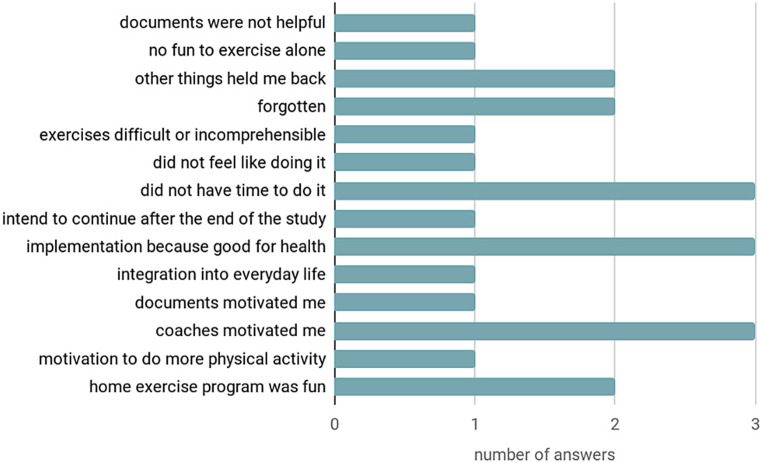
Feedback on the home exercise program (multiple answer options)

The implementation of the home exercise program varied widely. Overall, 29% of the participants never performed the training at home. The three-page home exercise document with description and pictures of the exercises was clear and comprehensible for the participants and 43% of the participants indicated not having enough time to read the home exercise program (Fig. 2).

## Discussion

In the before-after comparison little changes in motivation were observed. The results show that contrary to expectations the motivation of the obese adolescents to move and to participate in the study was generally high. It was mainly influenced by the forms of identified and intrinsic motivation according to Ryan and Deci [16] that are of particularly high quality. The adolescents indicated to be active in sports and showed an interest in physical activity.

The motivation to participate in the sports program was strongly identified. This means that our adolescents with obesity were aware of the importance of exercise for their own health. This could also be an explanation for the high intrinsic motivation. The largely balanced gender ratio among the group leaders (three women, two men) could also have contributed to less body shaming and more physical well-being of our study group. The motivation to move was moderately influenced by external factors, and both the introjected and the extrinsic motivation could be reduced in the before-after comparison. Contrary to expectations, the results were generally positive when it came to the quality of the motivation to move. Other studies emphasized the increased intrinsic motivation of normal weight adolescents compared to obese ones [25]. Since the comparison group is missing in this study, no further statements can be made here.

Our participants hardly carried out the home exercise program. Although the group leaders motivated the adolescents and they were well aware of how important exercises are for their own health, lack of time was the main answer. This implies the assumption of a stressful everyday life already in adolescence or another prioritization in the organization of leisure time;however, digital technologies could be a successful way to improve acceptance compared to providing the program as a paper copy. The home exercise program should be available on the mobile phone at any time, exercise videos with the group leaders applicable for mobile phones, tablets or laptops could be another possibility. The online training sessions should be available at a fixed time schedule. In addition, each participant can decide whether to activate his or her own camera. Then siblings and parents could also participate in the training sessions to achieve a holistic family approach and to increase motivation and compliance of obese adolescents. A current randomized controlled pilot study for app-based exercise support in orthopedic rehabilitation showed that an exercise program significantly increased activity level and compliance compared to exercise documents on paper [26]. Online training is also a very good option with respect to coronavirus disease 2019 (COVID-19). School and associated obligations such as homework and studying for school examinations were an enormous stress factor for the teenagers. Hence, training offered during summer school holidays, might increase the training participation and is beneficial in terms of the corona pandemic when training sessions can be performed outside. Especially with this specific group of patients, rewards might help to motivate to move in trainings on site, at home or online. Therefore, rewards as motivational factor should be considered in the following study. It is interesting that almost one third of the patients had back pain. It is therefore important in the future to involve specific exercises to relieve and strengthen the back.

The importance and integration of behavioral therapy and psychotherapy, but also social work in relation to obesity therapy should be examined in more detail. Only a concept that contains different areas for behavior change makes sense for sustainable obesity treatment or prevention [6, 7]. Future studies should integrate ecosocial and social areas, such as migrant background, educational and socioeconomic status, and work-life balance. The promotion of volition as an implementation competence of health goals is important. Therapy based on the biopsychosocial model is successful if diagnosis and treatment are carried out in parallel at all relevant levels. This requires interdisciplinary cooperation between the health professions. Politicians must contribute to create social and eco-social conditions for health promotion of obesity prevention and therapy to be feasible in a sustainable manner. The research and improvement of the multimodal, multidisciplinary and biopsychosocial treatment approach should remain the focus in obesity therapy. The promotion of motivation for movement is essential.

### Strengths and limitations

A strength of the study is that an individualized training based on the SDT was investigated for the first time. Therefore, this pilot study might be the basis for future training sessions with this specific and vulnerable group. Training, tools and questionnaires were also examined. The results of MotiMove study will enable physiotherapists and movement experts to design and perform training for obese adolescents. Moreover, MotiMove forms the basis for the next study with a control group.

Another strength is that the local Viennese sports association came to the last training session and provided exercise programs related to the sports preferences of our study sample. These sports programs are available in most of the districts of Vienna and therefore promote an easy access for our target group.

Limitations of the MotiMove study are the small sample size, the lack of a control group and the relatively short intervention period. Nevertheless, the MotiMove study and the results respond to a very current topic as prevalence of obesity still increases. Due to the actual situation around COVID-19, obese children and adolescents are now more at risk of stigmatization than before as socially marginalized group with an increased hazard for metabolic and cardiovascular diseases, a low quality of life and low self-esteem. For example, physical education is currently limited in Austrian schools and government measures like home schooling have an especially negative effect on children of families with low socioeconomic status. Social inequality is exacerbating. Movement programs, additionally to physical education in schools focused on promotion of motivation and therefore self-efficacy, are highly relevant as an answer for serious sociopolitical matters.

It is questionable whether a longer study period is effective for these patients. This study was able to show that obese adolescents did not always show up for training due to stress at school, lack of time management and ongoing private activities. The adolescents have no additional effort to drive by public transport to the training. Therefore, a weekly additional online live training can be discussed.

The fact that all participants had a migrant background might be a selection bias. It must be mentioned that almost all patients in the outpatient clinic of obesity in Vienna have a migration background. The question arises how effective its implementation was, whereby a differentiation in the quality of the lessons is objectively difficult to measure. All group leaders were trained and followed the same procedure.

## Conclusion

In this pilot study, there were little changes in the before-after comparison in motivation for movement of obese adolescents. Contrary to expectations, the motivation to move and to participate in the study was generally high. Digital technologies could be a successful way to further increase motivation and compliance for the home exercise program. This MotiMove study is a basis for future research programs and empower physiotherapists and movement experts to develop and implement training programs for obese adolescents and children.

## Supplementary Information

Home exercise program

## References

[1] Abarca-Gómez L, Abdeen ZA, Hamid ZA, Abu-Rmeileh NM, Acosta-Cazares B, Acuin C (2017). Worldwide trends in body-mass index, underweight, overweight, and obesity from 1975 to 2016: a pooled analysis of 2416 population-based measurement studies in 128·9 million children, adolescents, and adults. Lancet.

[2] Chooi YC, Ding C, Magkos F (2019). The epidemiology of obesity. Metabolism.

[3] Bischoff SC (2017). Adipositas: Neue Forschungserkenntnisse und klinische Praxis.

[4] Bundesministerium für Gesundheit und Frauen (BMGF). Childhood Obesity Surveillance Initiative (COSI); Bericht Österreich 2017. 2017. https://broschuerenservice.sozialministerium.at/Home/Download?publicationId=521. Accessed 5 Oct 2020.

[5] World Health Organization. Obesity. 2020. https://www.who.int/westernpacific/health-topics/obesity. Accessed 5 Oct 2020.

[6] Jenull B, Trapp E-M (2015). Biopsychosozialer Ansatz der Adipositas im Kindes- und Jugendalter. Psychotherapeut.

[7] Zeiher J, Varnaccia G, Jordan S, Lange C (2016). Was sind die Einflussfaktoren kindlicher Adipositas?: Eine Literaturübersicht im Rahmen des Projekts „Bevölkerungsweites Monitoring adipositasrelevanter Einflussfaktoren im Kindesalter“. Bundesgesundheitsblatt.

[8] Gerber M (2015). Pädagogische Psychologie im Sportunterricht: Ein Lehrbuch in 14 Lektionen.

[9] Lian Q, Su Q, Li R, Elgar FJ, Liu Z, Zheng D (2018). The association between chronic bullying victimization with weight status and body self-image: a cross-national study in 39 countries. PeerJ.

[10] Pont SJ, Puhl R, Cook SR, Slusser W, Section On Obesity, The Obesity Society (2017). Stigma experienced by children and adolescents with obesity. Pediatrics.

[11] Graf C, Beneke R, Bloch W, Bucksch J, Dordel S, Eiser S (2013). Vorschläge zur Förderung der körperlichen Aktivität von Kindern und Jugendlichen in Deutschland: Ein Expertenkonsens. monatsschr Kinderheilkd.

[12] García-Hermoso A, Ramírez-Vélez R, Saavedra JM (2019). Exercise, health outcomes, and pædiatric obesity: a systematic review of meta-analyses. J Sci Med Sport.

[13] Messerli-Bürgy N, Horsch A, Schindler C, Boichat A, Kriemler S, Munsch S (2019). Influence of acute physical activity on stress reactivity in obese and normal weight children: a randomized controlled trial. Obes Facts.

[14] Sirico F, Bianco A, D’Alicandro G, Castaldo C, Montagnani S, Spera R (2018). Effects of physical exercise on Adiponectin, Leptin, and inflammatory markers in childhood obesity: systematic review and meta-analysis. Child Obesity.

[15] McGrane N, Cusack T, O’Donoghue G, Stokes E (2014). Motivational strategies for physiotherapists. Phys Ther Rev.

[16] Ryan RM, Deci EL (2018). Self-determination theory: basic psychological needs in motivation, development, and wellness.

[17] Ziviani J, Poulsen A, Cuskelly M (2012). The art and science of motivation: a therapist’s guide to working with children.

[18] Fenton SAM, Duda JL, Appleton PR, Barrett TG (2017). Empowering youth sport environments: Implications for daily moderate-to-vigorous physical activity and adiposity. J Sport Health Sci.

[19] Baumann S (2016). Psychologie im Jugendsport: Der Einfluss der Pubertät – Die Auswirkungen auf das Lernen – Die Rolle des Trainers.

[20] Behzadnia B, Adachi PJC, Deci EL, Mohammadzadeh H (2018). Associations between students’ perceptions of physical education teachers’ interpersonal styles and students’ wellness, knowledge, performance, and intentions to persist at physical activity: a self-determination theory approach. Psychol Sport Exerc.

[21] Cuevas R, Ntoumanis N, Fernandez-Bustos JG, Bartholomew K (2018). Does teacher evaluation based on student performance predict motivation, well-being, and ill-being?. J Sch Psychol.

[22] Silva MN, Marques MM, Teixeira PJ (2014). Testing theory in practice: the example of self-determination theory-based interventions. Eur Health Psychol.

[23] Kromeyer-Hauschild K, Wabitsch M, Kunze D, Geller F, Geiß HC, Hesse V (2001). Perzentile für den Body-mass-Index für das Kindes- und Jugendalter unter Heranziehung verschiedener deutscher Stichproben. Monatsschr Kinderheilkd.

[24] Kohake K, Lehnert K (2018). Konstruktion eines Fragebogens im Rahmen der Selbstbestimmungstheorie der Motivation im außerschulischen Sport im Kindesalter. Ger J Exerc Sport Res.

[25] Power TG, Ullrich-French SC, Steele MM, Daratha KB, Bindler RC (2011). Obesity, cardiovascular fitness, and physically active adolescents’ motivations for activity: A self-determination theory approach. Psychol Sport Exerc.

[26] Bui T, King C, Llado A, Lee D, Leong G, Paraparum A (2019). App-based supplemental exercise during inpatient orthopaedic rehabilitation increases activity levels: a pilot randomised control trial. Pilot Feasibility Stud.

