# Supplementary material for: Impact of self-determination theory in a physiotherapeutic training: A pilot-study on motivation for movement of obese adolescents
Source: Wien Klin Wochenschr. 2021 Apr 9;134(5-6):208–14. doi: 10.1007/s00508-021-01849-4 (PMC8034041; doi:10.1007/s00508-021-01849-4)
Supplement: Supplementary file 1 — Home exercise program [file 508_2021_1849_MOESM1_ESM.pdf]

MotiMove Home Exercise Program

Dear participants,

This is your home training program. Please arrange to complete the exercises individually **at least once a week for 15 to 20 minutes**. Previously, you will go through all of the exercises in the training sessions with your trainer. If nothing else is indicated, repeat each exercise **ten times** and complete **three sets**. You might want to switch on some music or have another person joining you to make the exercises easier. So: **Let‘s get started!**

Squats

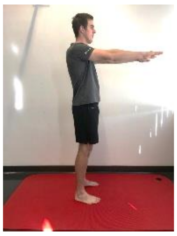

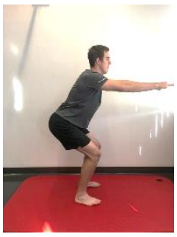

Stand up and put your arms straight in front of you. Your feet are shoulder width apart, your toes face forward.

Bend your knees by lowering your buttocks. Stay here for 3 cycles of breath and then return to the starting position. Be careful …

- … to keep your back straight
- … to form a line with your hips, knees and ankles
- … not to let your knees extend over your toes

One Leg Stand

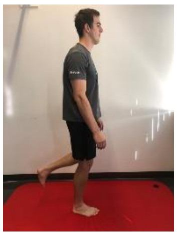

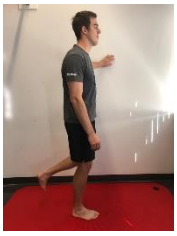

Stand upright on both legs and activate your belly muscles by slightly tucking in your pelvis. Then lift up to the **one leg stand**. Be careful …

- … that your heel and your forefoot remain in touch with the ground
- …to keep your hips, knees and ankles in one line

Try to remain in this position three times on each side for at least 10 seconds.

Cat Cow Pose

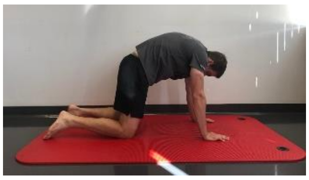

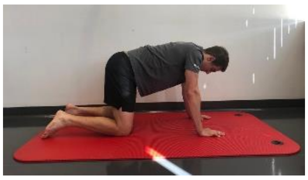

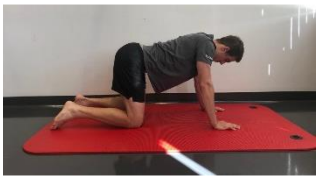

Place your hands and knees on the mat with your wrists directly under your shoulders and your knees under your hips. Keep your spine straight and ensure that your head is a prolongation of your spine. Keep your elbows slightly bent.

- **Cow pose:** inhale, lift your head and your breastbone and lower your belly.

Photos and Logo © Tobias Fürnsinn

- **Cat pose:** exhale and round your back by tucking your head and your pelvis towards the center of your body.
- Change your breathing rhythm between the two poses.

Variation: In cow pose, **lift one arm and the opposite leg** from the ground and **stretch them straight in front of you or backwards.** Slide your elbow and the opposite knee joint in front of your navel. Complete one side, then change to the other.

Bridging

Lay on your back and **bend your legs towards your buttocks.** Your arms rest next to your body. Tuck your pelvis in and start **lifting your buttocks and your spine** from the mat. Hold this position for 5 to 10 seconds and then lower your spine and your buttocks back to the mat.

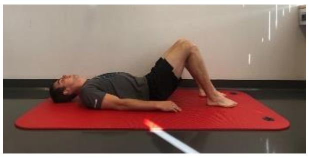

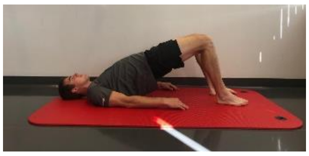

Jumping Jack

Stand upright with your feet around hip width apart, your toes face forward. Simultaneously raise your arms **out to your sides above your head** and **jump to spread your feet wide**. Now, come back to the starting position. Repeat this exercise for about 2 minutes. Be careful …

- …not to let your knees face inwards while jumping.

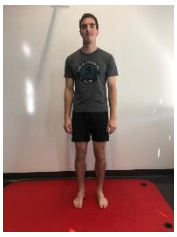

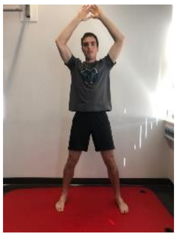

Day to day Recommendations

Here are some simple day to day advices to help **you to integrate physical activity into your daily life.**

- Take the stairs instead of the escalator or the elevator.
- Hop off public transport one station earlier and walk the rest of the way.
- Travel shorter distances by bike.
- Go for regular walks. Take a friend, a family member or your dog with you.
- Change your chair for a gymnastic ball from time to time.
- Make a conscious effort to stand up and walk around while talking on the phone.
- When brushing your teeth: Lift to the one leg stand and balance your weight back and forth from toes to heels.
- When waiting: Flex your buttocks, then release or just stand on one leg to train your sense of balance.

Photos and Logo © Tobias Fürnsinn

How many calories do I burn by…?

- …climbing 4 floors or stairs: about 19 kcal
- …30 min cleaning or tidying up: about 115 kcal
- …going for a walk for 15 min: about 60 kcal
- …20 min of power walking: about 200 kcal
- …60 min of water gymnastics: about 400 to 500 kcal
- …60 min of cycling: about 400 to 600 kcal
- …30 min of swimming: about 420 kcal
- …10 min of laughing: about 60 kcal **Keep on smiling!** ☺☺☺

Photos and Logo © Tobias Fürnsinn
